# Roles of CD38 in the Immune Response to Infection

**DOI:** 10.3390/cells9010228

**Published:** 2020-01-16

**Authors:** Estibaliz Glaría, Annabel F. Valledor

**Affiliations:** Department of Cell Biology, Physiology and Immunology, School of Biology, University of Barcelona, and Institute of Biomedicine of the University of Barcelona (IBUB), 08028 Barcelona, Spain; estiglari@gmail.com

**Keywords:** CD38, CD157, immune response, infection

## Abstract

CD38 is a multifunctional protein widely expressed in cells from the immune system and as a soluble form in biological fluids. CD38 expression is up-regulated by an array of inflammatory mediators, and it is frequently used as a cell activation marker. Studies in animal models indicate that CD38 functional expression confers protection against infection by several bacterial and parasitic pathogens. In addition, infectious complications are associated with anti-CD38 immunotherapy. Although CD38 displays receptor and enzymatic activities that contribute to the establishment of an effective immune response, recent work raises the possibility that CD38 might also enhance the immunosuppressive potential of regulatory leukocytes. This review integrates the current knowledge on the diversity of functions mediated by CD38 in the host defense to infection.

## 1. Introduction

The immune system is composed of a tightly regulated network of cells and molecules that cooperate to protect the organism from a diversity of dangerous agents. Compartmentalization of the immune system guarantees constant monitoring of tissues and controlled activation of the immune response to eliminate pathogens and other harmful agents and return to homeostasis. The innate immune system is essential for the initial containment of pathogens. Professional phagocytes such as macrophages and neutrophils are recruited toward the infection site to engulf and kill microorganisms. Dendritic cells internalize exogenous agents and migrate to the lymph nodes to trigger activation of the adaptive immune system. Upon activation by antigen presentation through major histocompatibility complexes (MHC), T cells either execute cytolysis on target infected cells (cytolytic T cells) or secrete cytokines to further modulate the type of immune response (helper T cells, Th). In addition, B cells can detect antigens on pathogens and differentiate to antibody-producing plasma cells [1].

CD38 is a multifunctional transmembrane protein that is widely expressed in immune cells. In lymphocytes, monocytes, macrophages, dendritic cells, granulocytes, and natural killer (NK) cells, the expression levels of CD38 on the cell surface depend on the stage of maturation and/or activation of the cell (reviewed in [2]). Since its discovery almost four decades ago [3], accumulated evidence indicates that CD38 plays important roles in various cell types in both physiological and pathological contexts. Early studies suggested that human CD38 can establish lateral associations with various membrane proteins/complexes, such as CD16 (in NK cells), the T cell receptor (TCR)/CD3 complex and CD4 (in T cells), membrane immunoglobulin (Ig) and the B cell co-receptor complex (CD19/CD81) (in B lymphocytes), and class II MHC (in monocytes) [4,5,6]. On the basis of such interactions, CD38 was proposed to potentially contribute to cell signaling from these complexes. In agreement with this notion, and despite the fact that CD38 contains a short cytoplasmic domain without signaling motifs, CD38 relocalized at the immunologic synapse in T cells upon TCR engagement, contributing to modulation of antigen-mediated T-cell responses [7]. In the same line, CD38 crosslinking decreased the threshold for B cell activation via the B-cell receptor (BCR) [8], suggesting its participation in BCR signaling. Human CD38 was also shown to bind a specific nonsubstrate ligand, CD31/PECAM-1, a member of the Ig superfamily that is highly expressed on the surface of different cell types, including endothelial cells [9]. Interference with CD38–CD31 interaction inhibited lymphocyte adhesion to endothelial cells.

In addition to receptor or co-receptor functions, CD38 also plays multiple roles derived from intrinsic enzymatic activities. At neutral pH, CD38 converts nicotinamide adenine dinucleotide (NAD) into ADP ribose (ADPR), cyclic ADPR (cADPR) and nicotinamide, whereas at acidic pH, CD38 uses NAD phosphate (NADP) to generate nicotinic acid adenine dinucleotide phosphate [10,11]. The enzymatic products of these reactions are calcium-mobilizing second messengers with relevant signaling consequences in diverse cellular contexts. Because the generation of ADPR and cADPR requires large consumption of NAD, CD38 is considered the major NAD glycohydrolase (NADase) in mammalian tissues [12]. In addition, CD38 can also catabolize the extracellular NAD^+^ precursors nicotinamide mononucleotide and nicotinamide riboside before they are transported into the cell for NAD^+^ biosynthesis [13].

The consequences of CD38 expression depend also on the ultrastructural configuration of the molecule and its location within the cell (reviewed in [14]). CD38 has been shown to exist as either monomeric [15], dimeric or even multimeric type II forms [16,17], displaying the catalytic site outside of the cell, and as a type III form with the catalytic site facing the cytoplasm [18]. Moreover, an intracellular pool of CD38 has been shown to be associated to mitochondrial and nuclear membranes (reviewed in [19]). In these configurations, CD38 could have access to both extracellular and intracellular NAD^+^. In addition, CD38 also exists as a soluble form, which is detectable in biological fluids [20].

Because of its abundant expression in immune cells, several studies have focused on the roles of CD38 in the immune response to infection. Both in vivo and in vitro models have been used in combination with CD38 deficiency, ligating/blocking antibodies or agonists/antagonists of CD38 activities in order to decipher the effects of CD38 in different cell types and infection settings. The aim of the present review is to provide an updated overview on the diversity of functions mediated by CD38 in the context of the host defense to infection.

## 2. CD38 Deficiency Results in Increased Susceptibility to Several Pathogens

Pathogenic bacteria are causative agents for a wide spectrum of infectious diseases. Bacterial infection causes tissue damage through different mechanisms, including the killing of infected host cells, the secretion of toxins and the induction of an exacerbated inflammatory response [21]. In vivo studies demonstrated that CD38 deficiency in mice conferred increased susceptibility to infection by several bacteria, namely *Listeria monocytogenes* (*L. monocytogenes*) [22], *Mycobacterium avium* (*M. avium*) [23] and *Streptococcus pneumoniae* (*S. pneumoniae*) [24,25], and the parasite *Entamoeba histolytica* (*E. histolytica*) [26].

In addition, CD38 was recently shown to be a transcriptional target of the nuclear receptor liver X receptor (LXR), which is activated by derivatives of cholesterol metabolism [27,28]. Pharmacological treatment with a synthetic LXR agonist ameliorated the clinical severity of *Salmonella* Typhimurium (*S.* Typhimurium)-infected mice and reduced the dissemination of the bacteria to the spleen in a CD38-dependent manner. Of note, the expression of CD38 in bone marrow-derived cells was required for the ameliorating effects of the LXR agonist [27].

Altogether, the observations in animal models indicate the importance of CD38 in the control of infection, raising its potential interest as a target for host-directed therapy against infection. An overview of mechanisms associated with the multifaceted nature of CD38 that modulate the establishment of an effective immune response is provided in the following sections.

## 3. CD38 Contributes to Pro-Inflammatory Phenotypes in Innate Immune Cells

Pathogens that overcome natural barriers of the body can be subsequently recognized by innate immune cells. Macrophages, neutrophils and dendritic cells detect pathogen-associated molecular patterns (PAMPs) through specialized receptors and initiate signaling cascades that lead to phagocytosis and production of inflammatory mediators [1].

**Figure 1:**
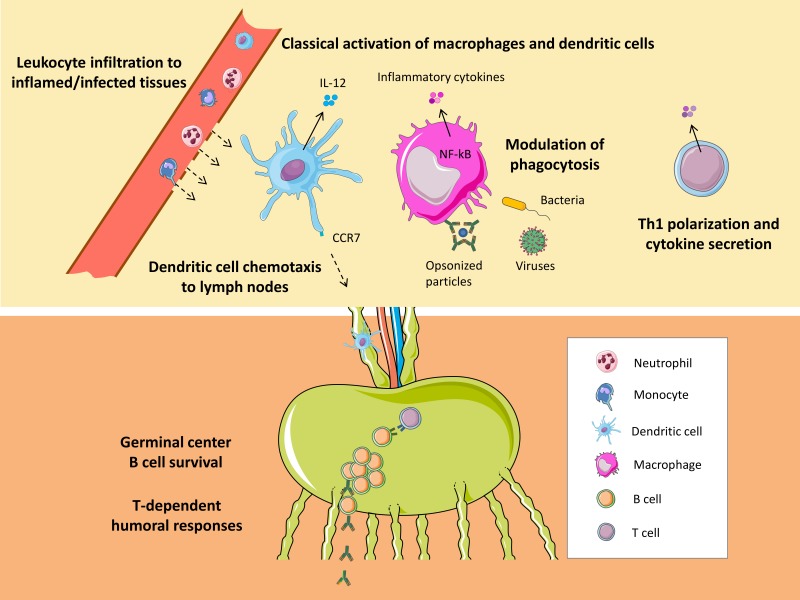
Summary of immunological roles of CD38 in the response to infection. Steps of the immune response to pathogens for which there are solid data involving the participation of CD38. Some elements in the image have been obtained from Smart Servier Medical Art.

Pro-inflammatory cytokines produced by the host, such as tumor necrosis factor alpha and interferon gamma (IFNγ), or the bacterial component lipopolysaccharide (LPS) induced the expression of CD38 in murine and human macrophages [22,27,29,30,31,32] and during maturation of dendritic cells [28,33]. Reciprocally, accumulated evidence suggests that CD38 helps sustain classical activation of macrophages and dendritic cells (Figure 1). In this sense, CD38 signaling upon ligation by monoclonal antibodies induced cytokine secretion in resting human monocytes [34] and enhanced interleukin (IL)-12 production in synergy with IFNγ in human dendritic cells [33]. The effects on monocytes were also observed upon CD38 interaction with CD31 [34]. Furthermore, the lack of functional CD38 expression or the selective interference with its receptor or enzymatic activities in myeloid cells resulted in reduced production of pro-inflammatory mediators in response to LPS [35,36] or to bacterial [27] or viral infection [37]. In macrophages, these effects correlated with inhibition of the activation of the NFκB signaling pathway [36].

## 4. CD38 Enzymatic Activities Regulate Leukocyte Infiltration to Infected/Inflamed Tissues

Several inflammatory mediators, including cytokines and chemokines, increase vascular permeability to facilitate sequential recruitment of immune cell types toward the site of infection [38]. Furthermore, molecules released by infectious agents are also recognized as chemoattractant signals for a number of infiltrating cells [39].

In addition to a direct role of CD38 signaling on pro-inflammatory myeloid cell activation, the enzymatic activities of CD38 and the subsequent generation of calcium-mobilizing second messengers are important for the recruitment of different leukocytes toward a number of chemotactic signals produced at the site of infection [24,40,41] (Figure 1). A decrease in neutrophil accumulation in *S. pneumoniae*-infected lungs was reported in mice lacking functional CD38 expression, in correlation with reduced in vitro migration of CD38-deficient neutrophils to the chemoattractant formylmethionyl-leucyl-phenylalanine [24,25]. Likewise, CD38 deficiency resulted in lower infiltration of innate leukocytes in the liver and spleen of *Listeria*-infected mice [22] and in delayed recruitment of neutrophils to livers infected with the parasite *E. histolytica* [26]. The intracellular calcium rise and the chemotactic response of murine neutrophils to formyl peptide receptor ligands was inhibited by the cADPR and ADPR antagonists 8-Br-cADPR and 8-Br-ADPR, respectively [40]. Similar results were obtained upon treatment with a NAD^+^ analog, N(8Br-A)D^+^, which can be converted to 8-Br-cADPR by the ADP-ribosyl cyclase activity of CD38. Furthermore, antagonistic analogs of cADPR and ADPR also blocked the chemotaxis of other leukocytes of human and murine origin to multiple chemoattractant signals, including inflammatory chemokines [40,41].

## 5. Multifaceted Roles of CD38 in Phagocytosis

Within infected tissues, phagocytosis is a major mechanism used by professional phagocytes to eliminate pathogens and dead cells [42]. Internalized bacteria are then killed and digested in specialized phagolysosomes.

CD38-deficient macrophages displayed impaired capability to phagocytose *L. monocytogenes* in vitro [22]. In experiments in which mice were first infected with *L. monocytogenes* and then injected with fluorescent latex beads, CD38^+^ inflammatory monocytes and neutrophils recovered from the liver had taken up more beads than their CD38^-^ counterparts [43], suggesting that CD38 activities could also facilitate unspecific engulfment.

CD38 also positively regulated phagocytosis of latex beads coated with IgG in the absence of PAMPs [44], a mechanism that is mediated by Fcγ receptors. CD38 was shown to be recruited to the forming phagosomes during internalization of IgG-opsonized particles by macrophages, with the catalytic domain oriented to the lumen and correlating with an increase in intracellular cADPR and calcium mobilization. The use of an antagonistic analog of cADPR or peritoneal macrophages from CD38-deficient mice impaired the phagocytosis of IgG-coated latex beads. Noteworthy, the environment usually found during the primary response to infection includes the presence of PAMPs and an inflammatory milieu that might be complemented later with antigen-specific antibodies if the adaptive immune system becomes activated. Thus, the role of CD38 in mediating phagocytosis of IgG-opsonized material might be of particular relevance once the adaptive immune response has generated antibodies against the pathogen (Figure 1).

In contrast with CD38 facilitating the internalization of *L. monocytogenes* and latex beads, treatment of macrophages with an LXR agonist limited the internalization of *S.* Thyphimurium, an effect that was largely dependent on functional CD38 expression [27]. Macrophages sensing *Salmonella* infection underwent both extensive dorsal accumulation of F-actin cytoskeletal structures and morphological changes that might facilitate the entry of invasive bacteria. Once inside, *Salmonella* uses the macrophage as a niche for replication and dissemination (reviewed in [45]). Interestingly, the inhibitory actions of LXR agonists on the internalization of *S.* Thyphimurium were counteracted by abundant NAD^+^ levels, but a cADPR antagonist had no significant impact, suggesting that LXR-induced CD38 might contribute to control *Salmonella* infection in macrophages mostly by reducing the levels of NAD^+^. In line with this notion, treatment with FK866, a highly specific inhibitor of nicotinamide phosphoribosyltransferase that impacts NAD^+^ biosynthesis, resulted in decreased macrophage infection.

## 6. The Chemotactic Response of Dendritic Cells Is Modulated by CD38 Activity

Myeloid dendritic cells play an important role at the crosstalk between innate and adaptive immune responses. Upon pathogen recognition, they undergo maturation and migrate to draining lymph nodes in order to present antigens and trigger specific T-cell activation [1]. Mature dendritic cells express a C-C chemokine receptor (CCR)7, which directs migration to lymph nodes following a gradient of the chemokines C-C chemokine ligand (CCL)19 and CCL21 (reviewed in [46]).

Several studies propose the participation of CD38 in dendritic cell chemotaxis mediated by CCR7 (Figure 1), although different mechanisms have been reported in murine and human cells. In this regard, CD38-deficient mice displayed defective migration of dendritic cells to draining lymph nodes and impaired T cell-dependent humoral responses [47]. In vitro, CD38-deficient dendritic cells had an intrinsic inability to mobilize calcium and migrate in response to CCL19 and CCL21, and cADPR antagonism impaired the chemotactic response of dendritic cells to such ligands [28,47]. Interestingly, agonists that activate LXRs enhanced the chemotactic activity of murine dendritic cells toward CCL19, and this activity was largely dependent on functional CD38 expression and cADPR production [28].

In human dendritic cells, CD38 up-regulated chemotaxis to CCR7 ligands in vitro, but this effect was not abrogated by a cADPR antagonist [35]. Although the role of additional CD38-produced calcium-mobilizing second messengers was not tested, antibodies that block CD38–CD31 interaction impaired human dendritic cell chemotaxis. Moreover, in that work, CD38 was shown to co-localize with CCR7, CD83 and CD11b in membrane microdomains known as lipid rafts, suggesting that lateral associations between these proteins could contribute to the migratory activity of human dendritic cells.

## 7. CD38 Activities in the Adaptive Immune System

An effective adaptive immune response is initiated through antigen recognition and clonal expansion of T and B lymphocytes in secondary lymphoid organs. Mature B cells that are stimulated by antigens and T cell-derived signals proliferate within germinal centers and then differentiate toward either antibody-secreting plasma cells or memory B cells [1]. Because human CD38 is highly expressed on both germinal center B cells and plasma cells, it has been extensively used as a marker of B cell activation (reviewed in [48]). CD38 ligation prevented apoptosis of human germinal center B cells [49], suggesting that CD38 signaling could play a role in the selection of B cells within the germinal center. Interestingly, localization within lipid rafts and association with the CD19 complex were required for CD38-mediated signaling in human B cells [50].

Noteworthy, contrary to the observations in the human system, CD38 was down-regulated in murine germinal center B cells and mature plasma B cells [49]. Despite these discrepancies, CD38-deficient mice showed an impaired humoral response to T-dependent antigens after primary and secondary immunizations [51] (Figure 1). Although defects in innate immune activation and in leukocyte migration could lead to the observed phenotype, several studies have shown that CD38 engagement directly affects B cell responses also in mice. For example, in mature B cells, CD38 crosslinking induced tyrosine phosphorylation-mediated signal transduction, resulting in B cell proliferation and IgM secretion [52]. Moreover, CD38 provided a strong costimulatory proliferative signal to LPS-activated B cells and enhanced the expression of CD86, suggesting that CD38 might also facilitate costimulatory interaction between activated B and helper T cells [53].

Antigenic activation results in the generation of effector T cells that will recirculate from secondary lymphoid organs to sites of infection. Although the receptorial function of CD38 toward CD31 was shown to facilitate human T cell adhesion to endothelial cells [9], and it could be responsible for weak leukocyte binding to the endothelium [54], a role for CD38 in recruitment of effector T cells to sites of infection remains elusive. Evidence suggests, however, that CD38 could modulate inflammatory gene expression in helper T cells as is the case with innate immune cells. In this sense, CD38 ligation in human T lymphocytes in vitro resulted in the secretion of several cytokines, including IFNγ, IL-6, granulocyte-macrophage colony-stimulating factor, and IL-10 [55]. In addition, splenocytes from CD38-deficient mice infected with *M. avium* secreted lower amounts of IFNγ and displayed Th2 polarization, in correlation with their compromised ability to limit mycobacterial burden within granulomata [23].

The involvement of lymphocyte CD38 in the host defense against viruses has been mostly studied in the context of human immunodeficiency virus (HIV) infection. Interestingly, the levels of CD38 expression associated with prognosis differ depending on the population analyzed (adults *versus* children) (reviewed in [56]). Overexpression of CD38 on lymphocytes is, in fact, a strong predictor of CD4^+^ T cell depletion in adult HIV-infected individuals (reviewed in [57]). Early after HIV infection, the expression of CD38 increased in CD4^+^ and CD8^+^ T lymphocytes [58,59,60], and high proportions of the CD8^+^CD38^+^ subpopulation were associated with progression to acquired immune deficiency syndrome in adults [61,62]. In cross-sectional studies, a marked decline in the abundance of CD8^+^ T cells expressing CD38 was detected in patients on stable antiretroviral treatment [63]. Moreover, high levels of CD38 expression on CD4^+^ T cells was also a marker of poor prognosis in adult HIV-infected individuals [64,65]. In contrast to the evidences in adults, high frequencies of CD8^+^CD38^+^ or CD4^+^CD38^+^ T cells in children were associated with favorable prognoses [66,67].

Several studies have tried to understand the functional role of CD38 in HIV infection. In different human CD4^+^ T cell lines, the levels of CD38 expression correlated negatively with viral loads and HIV-1-induced cell death [68,69]. In addition, nicotinamide, a major product of CD38 activity, reduced the rates of HIV-1-induced cell death in CD38^low^CD4^+^ T cell lines. Together, these studies suggested that CD38 expression could inhibit lymphocyte susceptibility to HIV-1 infection and that its enzymatic activity could be involved in enhancing the survival of infected CD4^+^ T cells. It was proposed that increased expression of CD38 could mediate recycling of nucleotides and protect from cell death induced by nucleotide depletion [56]. In addition, HIV-1 envelope glycoprotein gp120 enhanced the lateral association between CD38 and CD4 at the cell membrane [4]. Interestingly, human CD38 was shown to inhibit the binding of either purified gp120 or HIV-1 with CD4^+^ cells [68] by displaying a sequence homologous to the V3 loop of gp120 [70]. These observations suggested that CD38 could play an inhibitory role on HIV-1 attachment to cells by interfering with gp120–CD4 interaction. However, while such a protective role would help explain the association between high CD38 expression levels and favorable prognoses in HIV-infected children, it may not offer an advantage for the control of infection in adults. Indeed, an interesting speculation was provided in [56] by proposing that because T cells displaying high levels of CD38 in adults are mostly activated cells that express chemokine receptors frequently used for viral attachment and entry, the effects of CD38-mediated protection of viral binding to these cells would have a minor impact.

Another aspect to take into account is the fact that gp120 was also shown to promote the dynamic binding of human CD4^+^ T cells to endothelial cells in vitro, through a mechanism potentially involving CD38–CD31 interaction [71]. In those studies, gp120 increased the homing of a murine T cell line expressing CD4^+^ into the spleen and intestine and mesenteric lymph nodes, raising the possibility that CD38 could be involved in mediating T cell homing during HIV infection. Nevertheless, despite CD38 overexpression representing a marker of activation and of poor prognosis in HIV infection, whether CD38 enzymatic activities play a role in the pathogenicity of HIV infection in adults remains an open question [57].

Interestingly, in a controlled *Plasmodium falciparum* (*P. falciparum*) infection study in humans, an expansion of CD38^+^CD4^+^ T cells was also detected in the peripheral blood of infected individuals and their frequency inversely correlated with parasite burden [72]. These cells exhibited cytolytic potential and impaired production of IFN-γ, although it was not determined whether CD38 provides an advantage to these cells in the control of *P. falciparum* infection.

## 8. CD38 in Sepsis

In some cases, the host response to an invading pathogen leads to the development of sepsis, a pathological syndrome with a hyperreactive phase in which exacerbated inflammation causes organ dysfunction followed by immunosuppression [73]. A role for CD38 in LPS-induced acute kidney injury [36] was suggested using quercetin, a dietary flavonoid that inhibits the NADase activity of CD38 [74,75]. Quercetin reduced the infiltration of neutrophils and macrophages in the liver. In line with the role of CD38 in maintenance of a pro-inflammatory phenotype, macrophages that had recovered from quercetin-treated mice had lower levels of NF-kB signaling and inflammatory markers as compared to macrophages from vehicle-treated mice [36]. Flavonoids, however, may also use protective mechanisms that are independent of CD38 inhibition, as the contributing role of CD38 in tissue damage was not confirmed in CD38-deficient mice [76]. CD38 deficiency aggravated kidney injury upon LPS-induced sepsis, in correlation with increased expression of Toll-like receptor (TLR)4 in the kidney and pro-inflammatory cytokine production. In a separate model of sepsis, induced by cecal ligation in rats, CD38 expression and cADPR production increased in the central nervous system [77]. Blocking this pathway through lentiviral-mediated CD38 knockdown or cADPR antagonism protected the hippocampus from apoptosis, oxidative stress and morphological damages associated with sepsis. Taking these contrasting observations into consideration, additional studies are required in order to assess whether interference with selective CD38 activities represents a strategy for the amelioration of immunopathology induced by excessive inflammation.

An aspect to consider is that regulatory T cells play key roles in limiting excessive immune responses. In mice and humans, regulatory CD8^+^ T cells displaying immunosuppressive actions on CD4^+^ effector T cell proliferation expressed high levels of CD38 [78]. In vivo, CD8^+^CD38^high^, but not CD8^+^CD38^-^, T cells ameliorated the clinical severity of murine experimental autoimmune encephalomyelitis, suggesting that CD8^+^CD38^high^ T cells are potential inhibitors of excessive immune responses. Likewise, high levels of CD38 expression on CD4^+^ regulatory T cells also correlated with superior suppressive activity [79]. These results raise interest in exploring whether any of the activities mediated by CD38 exert a direct role on the mechanisms for immunosuppression used by these cells. Moreover, regulatory T cells are also highly responsible for the immunosuppression phase associated with sepsis. Patients undergoing the hyporesponsive phase of sepsis fail to eradicate invading pathogens and become highly susceptible to opportunistic infections. Therefore, the role of CD38 in the immunosuppressive phase of sepsis should also be investigated.

## 9. CD157, a CD38 Paralogue, Is Important for the Host Response to *Mycobacterium tuberculosis*

CD157/bone marrow stromal cell antigen 1 (BST1) is a CD38 paralogue that can be found as a glycosylphosphatidylinositol (GPI)-anchored protein on the membrane or as a soluble form. Human CD157 is highly expressed on neutrophils, monocytes/macrophages, plasmacytoid and follicular dendritic cells, and many other cell types. CD157 exerts NADase activity, which leads to the production of ADPR. In humans, CD157, in contrast to CD38, has very limited ADPR cyclase activity, whereas in mice, this activity may be biologically important. In addition, an alternatively spliced form of CD157 exists with no detectable NADase activity (reviewed in [80]).

CD157 locates primarily within lipid rafts in the cell membrane. Despite not containing intracellular domains, CD157 was able to interact with integrins CD18 and CD29 on human neutrophils and monocytes, forming supramolecular complexes that activated signaling pathways to modulate transendothelial migration and adhesion to extracellular matrix components [81,82]. A number of extracellular matrix proteins containing heparin-binding domains were indeed shown to be high affinity non-substrate ligands for CD157, including fibronectin and fibrinogen [81,83].

Although limited information is available on the role of CD157 in the host response to infection, recent studies indicated that CD157 is important for conferring host resistance to *Mycobacterium tuberculosis* (*M. tuberculosis*) [84]. The activity of macrophages at the infection site is indeed critical for the host defense against *M. tuberculosis*. Importantly, CD157 expression was selectively up-regulated in circulating monocytes and in lungs from patients with tuberculosis and decreased after effective antituberculosis chemotherapy. CD157 contributed to the macrophage bactericidal activity by facilitating TLR2-dependent production of reactive oxygen species, an important mechanism used for bacterial killing. Interestingly, the levels of soluble CD157 correlated with human monocyte-derived macrophage bactericidal activity and exogenous administration of this form restored the bactericidal capacity of CD157-deficient macrophages, which raises the possibility that soluble CD157 might have a potential use in host-directed therapy against tuberculosis.

CD157 deficiency also resulted in alterations in the development of specific B cell subclasses, along with partial impairment of either the systemic humoral response after immunization with thymus-independent antigens or the mucosa-associated humoral response upon immunization with cholera toxin, a thymus-dependent antigen [85].

## 10. Increased Risk of Infections in Immunotherapies Using Anti-CD38 Antibodies

Because of its high expression in plasma cells, CD38 has emerged as a suitable target for immunotherapy of multiple myeloma [86]. Daratumumab is an approved anti-CD38 monoclonal antibody used for the treatment of multiple myeloma. The mechanism of action of daratumumab is based on the elimination of tumor cells expressing high levels of CD38 through antibody-dependent cellular cytotoxicity and complement-dependent cytotoxicity. Recent studies have reported an increased risk of infection in multiple myeloma patients undergoing treatment with daratumumab, either as monotherapy or as combined therapy with other drugs, with some cases resulting in lethal sepsis [87,88,89,90,91,92,93]. Infections reported as drug-related adverse effects include opportunistic bacterial infections of the respiratory or urinary tracts (e.g., *S. pneumoniae*, *Pseudomonas aeruginosa*, *Staphylococcus aureus*, *Escherichia coli* and *Haemophilus influenzae*), exogenous viral infections (e.g., acute respiratory syncytial virus, human metapneumovirus and influenza A and B viruses) and viral reactivations (e.g., cytomegalovirus, herpes simplex virus and varicella-zoster virus). Furthermore, increased risk of infection was also observed in a study exploring the effects of daratumumab in patients with systemic light-chain amyloidosis [94] and in clinical trials using isatuximab, a separate anti-CD38 monoclonal antibody, for multiple myeloma [95,96]. Of note, many patients undergoing anti-CD38-based immunotherapy are in relapsed or refractory phases of the disease and have been heavily treated, which implies that immunosuppression derived from previous lines of treatment could influence the risk of infection. Nevertheless, adverse effects associated with infection were also reported in patients with newly diagnosed multiple myeloma who were ineligible for autologous stem cell transplantation and received daratumumab as treatment [97].

Infectious complications may derive from anti-CD38 immunotherapy also targeting CD38-positive immune subpopulations different from tumor plasma cells. In fact, daratumumab treatment was shown to reduce the numbers of NK cells and other CD38-expressing immune cells [91,98]. Therefore, although additional studies are required to further understand the basis of daratumumab-induced immunosuppression in some patients, the accumulated data show the importance of cells expressing CD38 in the defense against pathogens in humans, which is consistent with the different activities of CD38 in the control of infection described earlier in this review.

## 11. Emerging Perspectives

The rapid development of antimicrobial resistances across the world is a major threat to public health, which is why efforts are addressed toward the discovery of novel approaches based on host-directed therapy to fight drug-resistant pathogens [99]. Lessons learned from anti-CD38 antibody therapy suggest that depletion of subpopulations of immune cells expressing CD38 increase the susceptibility to infection. However, whether manipulation of specific CD38 activities represents an adequate strategy for host-directed therapy against infections requires further investigation. In this sense, many studies support the importance of CD38 in maintaining pro-inflammatory profiles in innate immune cells. However, recent work has raised the possibility that CD38 might also enhance the immunosuppressive potential of regulatory lymphocytes. Therefore, the relative contribution of such opposing actions needs to be carefully examined. To date, the impact of the absence of functional CD38 in the host response to infection has been studied using systemic CD38-deficient mice. However, to better understand the different roles of CD38 in the immune system, approaches using conditional knockout models are required.

In addition, most attempts at dissecting the involvement of different activities associated with the multifunctional nature of CD38 have been accomplished using either blocking or agonistic antibodies or chemical inhibitors. Whereas such approaches have provided valuable information, more sophisticated models incorporating genetic modifications that interfere with selective CD38-mediated activities (whether receptorial or enzymatic) might help gain perspective on the relative importance of each of these functions in the immune response.

Among the varied consequences of increased CD38 expression, the implications of a substantial decline in NAD^+^ levels on the inflammatory response and the outcome on the course of infection offer an open area for exploration. Different studies have indeed generated discrepancies in regard to the effects of NAD^+^ depletion on inflammatory pathways. For example, a decrease in intracellular NAD^+^ through different mechanisms correlated with activation of the inflammasome in murine macrophages and the administration of exogenous NAD^+^ counteracted these effects [100]. In contrast, decreasing the levels of cellular NAD^+^ in human monocytes by using FK866 resulted in reduced TLR4 signal transduction and the inflammatory response to LPS [101]. Group A *Streptococcus* bacteria benefit, in fact, from the use of a molecule with NADase activity that inhibits inflammasome-dependent interleukin 1β release from infected macrophages [102]. Moreover, many bacteria use exogenous NAD to maintain their NAD turnover and limit the use of energy for NAD biosynthesis. The most drastic example is provided by the genus *Haemophilus*, which includes several pathogenic bacterial species that completely depend on exogenous NAD^+^ because they are not able to synthesize or recycle this molecule (reviewed in [19]). Therefore, the role of CD38 NADase activity in host cells as a protective mechanism against this type of pathogen deserves attention.

Apart from its cell surface and soluble forms, CD38 has also been identified within exosomes derived from HIV-1-infected lymphocytes [103]. Exosomes are extracellular vesicles that are secreted by the cellular endosomal compartment, and their cargos can change markedly during infection [104]. Exosomal cargos have been shown to influence different aspects of the host response, including the immune response to infection and the pathogenesis of sepsis [104,105]. Despite the fact that exosomal CD38 retains enzymatic activity [106], its role in extracellular NAD^+^ depletion and exosomal-mediated intercellular communication has not been characterized.

In conclusion, despite considerable knowledge existing on the diverse roles of CD38 in the immune response, new (and more sophisticated) approaches are required in order to determine the consequences of targeting specific CD38-mediated activities during the host response to infection.
